# Case report: A novel variant in *SLC25A46* causing sensorimotor polyneuropathy and optic atrophy

**DOI:** 10.3389/fneur.2022.1066040

**Published:** 2022-12-12

**Authors:** Louise Sloth Kodal, Sophia Hammer-Hansen, Sonja Holm-Yildiz, Karen Grønskov, Helena Gásdal Karstensen, Tina Dysgaard

**Affiliations:** ^1^Department of Neurology, Copenhagen Neuromuscular Center, Rigshospitalet, University of Copenhagen, Copenhagen, Denmark; ^2^Department of Clinical Genetics, Rigshospitalet, University of Copenhagen, Copenhagen, Denmark

**Keywords:** hereditary neuropathy, whole exome sequencing, polyneuropathy, optic atrophy, case report

## Abstract

*SLC25A46* is a mitochondrial protein involved in mitochondrial dynamics. Recently, bi-allelic variants have been identified as a pathogenic cause in a spectrum of neurological syndromes. We report a novel homozygous *SLC25A46* variant in two siblings, originating from Iraq. Both presented with optic atrophy and varying neurological symptoms. The neurological examination and nerve conduction studies were consistent with sensorimotor polyneuropathy, one having mild polyneuropathy and the other pronounced polyneuropathy. The cases illustrate the disease spectrum and provide substantial information to the knowledge of polyneuropathy caused by *SLC25A46* variants. It further highlights the diagnostic potentials of whole exome sequencing which can improve future understanding of disease mechanisms.

## Introduction

*SLC25A46* is a part of the Solute Carrier 25 (SLC25) family of transporters (1), a large family of more than 50 transporters varying in both size and transported substrates. The specific function is unknown, but it is thought to function as a transport protein in the outer mitochondrial membrane interacting with the inner membrane thereby contributing to mitochondrial dynamics involved in fusion and fission (2). Only a few patients with biallelic pathogenic variants in *SLC25A46* have been described in the literature. Patients can present with either Leigh syndrome (3), progressive myoclonic ataxia (4), or optic atrophy (OA), and limb spasticity (5). Only a few patients have been described with OA and axonal peripheral neuropathy (PN) due to variants in *SLC25A46* (6), and only one patient with OA, PN, and ataxia has been reported (7).

In this case report, we present the medical history and clinical and molecular genetic findings of two sisters, both presenting with decreased vision in varying degrees since childhood and varying PN symptoms due to homozygosity for a novel *SLC25A46* variant.

## Case description

Two sisters aged 26 (Patient A) and 34 (Patient B) were referred for genetic evaluation and diagnostic clarification due to childhood-onset optic atrophy.

### Family history and molecular genetics

The patients were originally from Iraq and of consanguineous parents. Patients A and B had seven siblings of which a brother also had optic atrophy since childhood and one sister experienced problems with balance.

**Figure 1:**
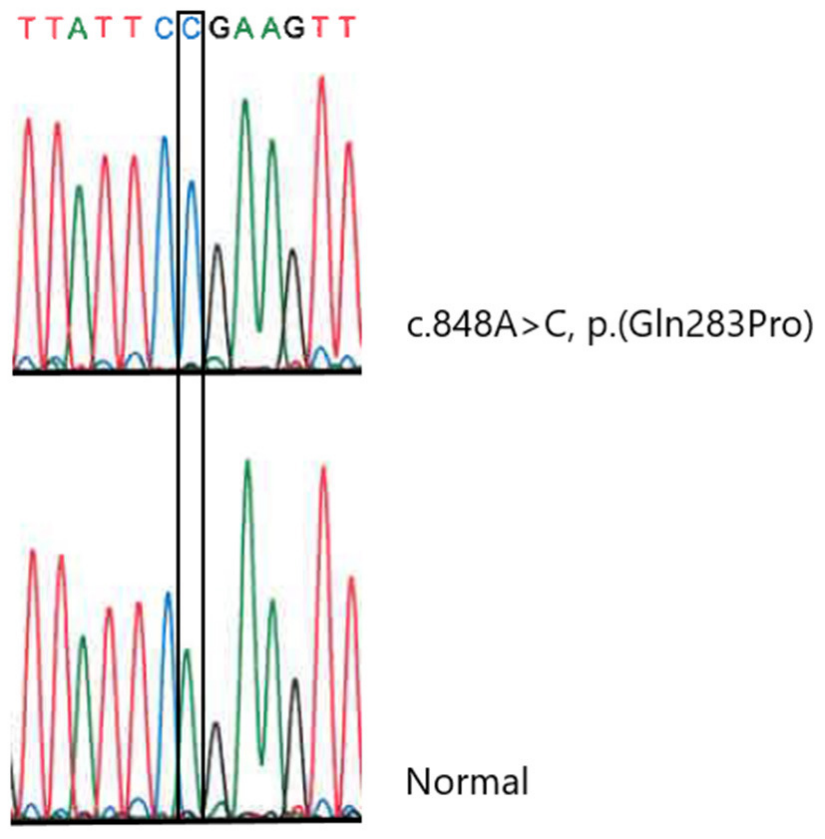
Sanger sequencing confirming the *SLC25A46* variant (NM_138773.3:c.848A>C, p.Gln283Pro).

**Figure 2:**
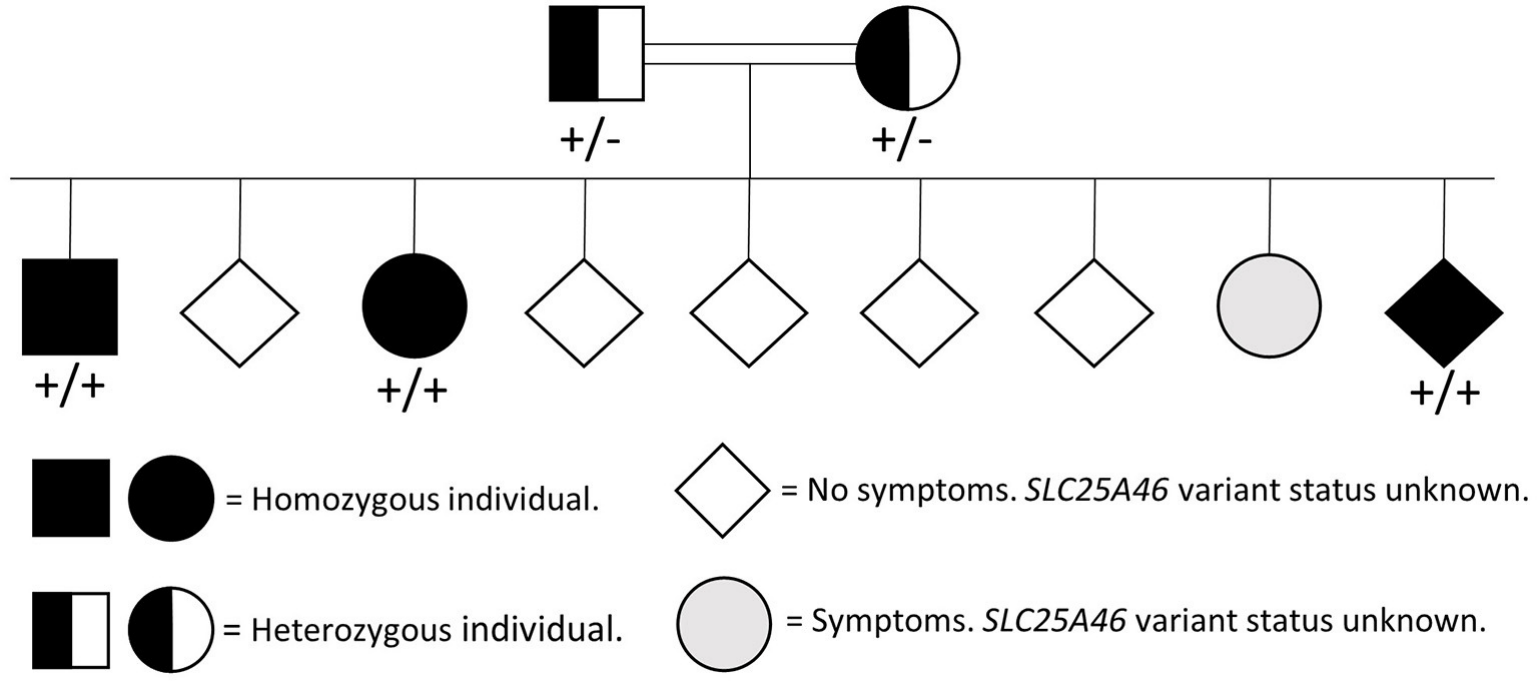
Pedigree showing the presence of the *SLC25A46* variant (NM_138773.3:c.848A>C, p.Gln283Pro) within the family.

Molecular genetic analysis of *OPA1* using gene-panel sequencing (NGS) and multiplex ligation-dependent probe amplification (MLPA) as well as PCR and Sanger sequencing of three mitochondrial variants [ND1 (m.3460G>A), ND4 (m.11778G>A), and ND6 (m.14484T>C)] was normal. Therefore, whole-exome sequencing (trio analysis with patient A as the proband) was performed revealing homozygosity for a novel *SLC25A46* variant [GRCh37, chr5:110,097,073, NM_138773.3:c.848A>C, p.(Gln283Pro)] in the proband, which was confirmed by Sanger Sequencing (Figure 1). Subsequently, patient B, as well as the affected brother were found to be homozygous for the variant. The variant has been reported in ClinVar in an affected individual (Accession number VCV001679272.1) and has not been reported in healthy controls [Genome Aggregation Database (version 2.1.1) or the UK biobank]. *In silico* models categorize the variant as pathogenic (CADD = 26, REVEL:0.758). A different missense variant at the same position (p.Gln283His) has been reported but has been classified as a variant of unknown significance in ClinVar. The variant was therefore classified as likely pathogenic according to ACMG guidelines for variant classification (PS4_sup; PM2_sup, PM3_sup, PP1_mod, PP3_sup) (8). The genetic testing of relatives was challenged and timely as some relatives lived abroad and contact was scarce. Both parents were tested and found to be heterozygous carriers and a symptomatic sibling was found to be homozygous (Figure 2).

Following genetic diagnosis, the two sisters were referred for neurological evaluation. The other sibling did not wish for further clinical workup.

### Patient A

A 26-year-old woman was seen in the Neuromuscular Clinic after referral. The patient was diagnosed with optic atrophy around the age of 13 years with symptoms since the age of 10 years but had not experienced progression in adult life. Patient A described herself as clumsier compared to her peers in childhood. The patient had since the age of 15 experienced sensibility disturbances lateral in the upper extremities and tingling and prickling sensation in the lower extremities and a general “weakness” when carrying heavy objects as well as impaired balance. She experienced a progression of sensibility disturbances and balance throughout the years (For timeline, see Supplementary material 2).

She had until recently worked as an ergo therapist and had now started a Master's degree. She experienced some cognitive symptoms with slightly impaired memory especially concerning numbers without worsening throughout the years. She was otherwise healthy and did not have diabetes or any use of medication except contraceptives. Patient A did not have any children yet but had thoughts about whether potential children would inherit the patient's symptoms. This had been the main concern for Patient A since adolescence and she had a wish for diagnostic clarification.

Neurological evaluation revealed diffuse slightly decreased muscle strength in the right hand (the patient was righthanded), impaired pinprick, and light touch sensations lateral on both arms. The Utah Early Neuropathy Scale (UENS) score was 0 out of a maximum of 42 points. A slightly unsteady gait when walking as on a tightrope was seen. Otherwise, the examination was normal.

Laboratory screening for treatable causes of neuropathy was normal including normal HbA1c. A nerve conduction study (NCS) showed reduced sensory nerve action potential in the median (up to the wrist) and sural nerves. No certain response was found in the peroneal nerves. Motor nerve studies were normal except for slowed conduction velocity in both ulnar nerves. The NCS was consistent with a predominantly sensory PN (Table 1). Cerebral MRI was normal.

**Table 1:** Nerve conduction studies.

**Nerve**	**Patient A's values**	**Interpretation**	**Patient B's values**	**Interpretation**
**Median L/R**				
Amplitude (μV)	NA/8.1	Abnormal	NA/2.3	Abnormal
CV (m/s)	NA/58	Normal	NA/52	Normal
**Ulnar L/R**				
Amplitude (μV)	15.1/17.3	Normal	NA	
CV (m/s)	57/57	Abnormal	NA	
**Sural L/R**				
Amplitude (μV)	1.7/1.2	Abnormal	%/%	
CV (m/s)	50/52	Normal	%/%	No certain response
**Tibial L/R**				
Amplitude (μV)	19.1/16.5	Normal	%/1.0	No certain response on left side. Severely reduced amplitude and CV on right side.
CV (m/s)	46/44	Normal	%/39	

CV, conduction velocity; L/R, left/right; NA, not applicable. %, no certain response to stimuli.

The patient was seen by a neuro-ophthalmologist where bilateral optic atrophy was confirmed and decreased vision was found (vision 6/12–6/18). The patient was recommended glasses and yearly follow-ups.

The patient was referred to the cardiology department, where an electrocardiogram (ECG), Holter monitoring, and heart ultrasound were performed. Holter monitoring and echocardiogram were normal with an ejection fraction (EF) of 60% and a global longitudinal strain (GLS) of 19. ECG was with abnormal unspecific changes and the patient is followed at a local cardiology clinic with annual controls including Holter.

No medical treatment exists for polyneuropathy due to an *SLC25A46* variant. Patient A was offered a referral to physiotherapy but did not feel a need for it.

One year later, the patient's ophthalmological, cardiological, and neurological symptoms were stationary. The patient is still followed yearly at the Neuromuscular Clinic as well as the Department of Cardiology and the Department of Ophthalmology.

### Patient B

A 34-year-old woman described herself as healthy except for a medical history of migraine with aura and decreased vision since early childhood. In school, she had problems reading what the teacher wrote on the blackboard and could not read books. She did not experience any problems with balance or gait and was a fast runner compared to her peers in school.

At the age of 25 years, she experienced increasing problems with balance and a tendency to have leg cramps (for timeline, see Supplementary material 2). Further, she experienced tinnitus with unknown debut without hearing loss. She had no complaints of sensibility disturbances or decreased muscle strength. The balance problems had increased slowly over the last 10 years, but her primary complaint was impaired vision. She was trained as a masseuse but is currently unemployed.

The cranial nerve examination was unremarkable except for a divergent eye axis and impaired vision with the ability to count fingers in half a meter's distance and the inability to read at a normal distance. The neurological examination of the upper extremities was normal with intact sensibility, motor function, and reflexes. No ataxia was found in the upper extremities. Hammertoes were observed and the neurological examination of the lower extremities revealed impaired vibration and position sense up to ankles, discrete sensory ataxia when performing the heel–knee–shin test, and decreased deep tendon reflexes in both ankles. The UENS score was 8. Romberg's sign was positive, and the gait was wide based on the ability to walk on a tightrope.

Blood screening showed a slightly increased kappa-free light chain test, 23.2 (reference interval 3.3–19.4), normal kappa/lambda ratio, and was interpreted without clinical significance. HbA1C was normal.

**Figure 3:**
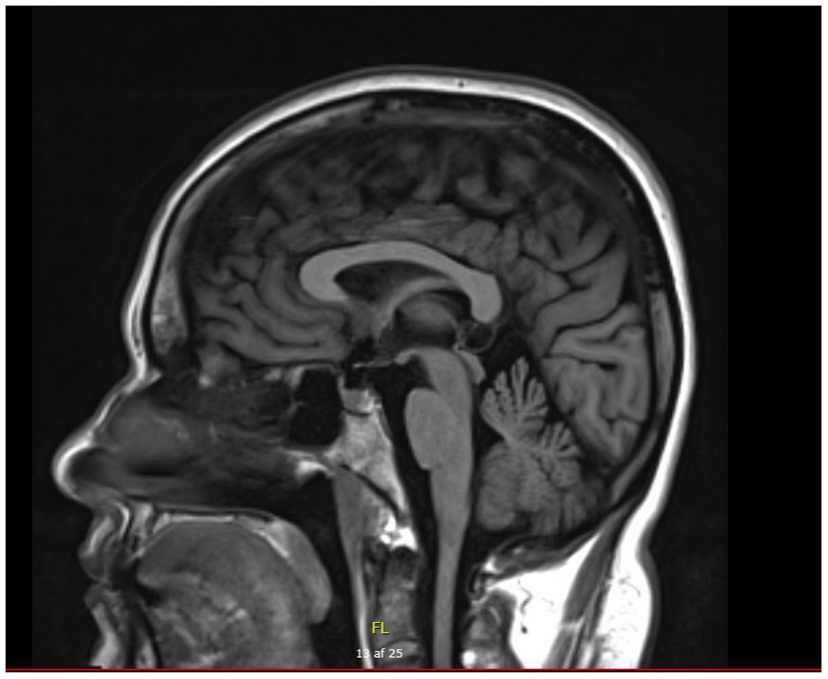
Cerebral MRI of patient B with cerebellar atrophy involving vermis.

A nerve conduction study showed reduced amplitude in median nerves, loss of response in sural nerves, and severely reduced and no certain motor response in right and left tibial nerves. The NCS was consistent with a pronounced axonal sensory-motor PN (Table 1). Cerebral MRI showed slight vermis atrophy in the cerebellum (Figure 3). Otherwise, the cerebral MRI was normal.

Ophthalmological examination found optic atrophy consistent with the previous OA diagnosis and significantly impaired vision (vision 6/60). The patient uses an enlarging TV system (CCTV).

ECG and echocardiography were normal with EF at 60% and GLS at 21. Audiological testing was performed because of the unspecific tinnitus, and normal hearing was found with an audiometry discrimination score of 96/96. The patient was referred to physiotherapy due to impaired balance and ataxia.

One year later the neurological symptoms as well as the neurological examination were stationary. Patient B is still followed in the neuromuscular clinic with yearly controls as well as yearly controls at the Department of Cardiology and the Department of Ophthalmology.

## Discussion

We report two patients with optic atrophy and polyneuropathy of varying degrees.

The patients had clinically been diagnosed with optic atrophy since childhood but did not have a genetic diagnosis for several years. The diagnosis was challenged as the patients were originally from Iraq and came to Denmark later in life which enhance cultural and language barriers. Furthermore, the family was spread across countries, and contact with some relatives was scarce. Patient A was initially tested for the gene *OPA1*, a more common gene related to hereditary optic atrophy. Due to a great wish for diagnostic clarification whole exome sequencing was performed and the patients were found to be homozygous for a novel, likely pathogenic *SLC25A46* variant (NM_138773.3:c.848A>C, p.Gln283Pro). Both parents were heterozygous carriers, and a symptomatic sibling was found to be homozygous for the *SLC25A46* variant.

Recently, the *SLC25A46*-associated PN has been classified as Charcot–Marie–Tooth Disease Type 6B, CMT6B, (9) and different variants in *SLC25A46* have been identified in different neurological syndromic forms with optic atrophy and axonal PN in four unrelated families in the United Kingdom, Palestine, Pakistan, the United States, and Italy (4, 7). However, the different phenotypes and clinical presentations including prognosis are still uncertain.

Patient A presented with mild OA at the age of 10 and a mild predominately sensory PN was found on ENG consistent with the neurological examination and the history of mild symptoms. Patient B presented with OA in early childhood and progressive balance impairment from the age of 25. A pronounced sensory-motor PN was found at the neurological examination and confirmed by nerve conduction studies. A cerebral MRI scan showed cerebellar atrophy. The cases illustrate the spectrum of the disease and confirm the previously described phenotypes in other variants of *SLC25A46*.

Patient A had a great wish for genetic testing and diagnostic clarification. She expressed concern about the disease and the risk of potential future children inheriting the disease. After investigation and diagnostic clarification, the patient was informed about the expected recessive trait and that Patient A's potential children will be carriers, but that the likelihood of symptomatic disease is very small with an unrelated partner. Patient B's main concern was visual impairment, and the diagnostic genetic clarification did not bring any further information for the patient as there is currently no treatment for the disease.

Our findings and genetic evaluation make the novel *SLC25A46* variant likely pathogenic and the varying phenotype with optic atrophy and polyneuropathy is supported by literature on other variants in *SLC25A46* where different neurological syndromes have been described (3–7).

Whole-exome sequencing can give diagnostic clarification and answers to patients even though the phenotype and prognosis may still be uncertain. Furthermore, it can improve understanding of disease mechanisms associated with polyneuropathy and CMT, which is needed to address relevant therapeutic areas.

## Conclusion

We present two sisters with varying polyneuropathy symptoms and symptomatic optic nerve atrophy due to a novel *SLC25A46* variant. The relatively late polyneuropathy diagnosis despite a known history of OA and consanguineous family history highlights the importance of thorough investigation as well as the diagnostic challenges in young patients. It further highlights the possibilities of whole exome sequencing which can improve future understanding of disease mechanisms.

## Data availability statement

The datasets presented in this article are not readily available because of ethical and privacy restrictions. Requests to access the datasets should be directed to the corresponding author.

## Ethics statement

Written informed consent was obtained from the individual(s) for the publication of any potentially identifiable images or data included in this article.

## Author contributions

LK: writing an initial manuscript draft, clinical examination, and manuscript editing and preparation. SH-H: clinical genetic workup and counseling, genetic data, and manuscript editing. SH-Y: clinical examination and manuscript editing. TD: conceiving the idea, supervising clinical examination and investigation, and editing the manuscript. KG: genetic data and manuscript editing. HK: genetic data and manuscript editing. All authors contributed to the article and approved the submitted version.

## Supplementary material

Supplementary material 1 Completed CARE checklist submitted as a supplementary file.

Supplementary material 2 Timeline of historical and current information for Patient A and Patient B.
