# Supplementary material for: Case report: A novel variant in SLC25A46 causing sensorimotor polyneuropathy and optic atrophy
Source: Front Neurol. 2022 Dec 12;13:1066040. doi: 10.3389/fneur.2022.1066040 (PMC9790965; doi:10.3389/fneur.2022.1066040)
Supplement: Supplementary material 1 — Completed CARE checklist submitted as a supplementary file. [file Data_Sheet_1.PDF]

CARE
case report guidelines

CARE Checklist of information to include when writing a case report

(cc) BY-NC-ND

✓

Topic	Item	Checklist item description	Reported on Line
**Title**	1	The diagnosis or intervention of primary focus followed by the words "case report"	1-2
**Key Words**	2	2 to 5 key words that identify diagnoses or interventions in this case report, including "case report"	13-14
**Abstract (no references)**	3a	Introduction: What is unique about this case and what does it add to the scientific literature?	25-28
	3b	Main symptoms and/or important clinical findings	21-23
	3c	The main diagnoses, therapeutic interventions, and outcomes	23-25
	3d	Conclusion—What is the main "take-away" lesson(s) from this case?	25-28
**Introduction**	4	One or two paragraphs summarizing why this case is unique (**may include references**)	39-41
**Patient Information**	5a	De-identified patient specific information.	43-44
	5b	Primary concerns and symptoms of the patient.	68-80+99-107
	5c	Medical, family, and psycho-social history including relevant genetic information	46-64
	5d	Relevant past interventions with outcomes	NA
**Clinical Findings**	6	Describe significant physical examination (PE) and important clinical findings.	81-83+108-115
**Timeline**	7	Historical and current information from this episode of care organized as a timeline	Figure 2
**Diagnostic Assessment**	8a	Diagnostic testing (such as PE, laboratory testing, imaging, surveys).	49-64+81-95+108-126
	8b	Diagnostic challenges (such as access to testing, financial, or cultural)	62-64
	8c	Diagnosis (including other diagnoses considered)	49-64
	8d	Prognosis (such as staging in oncology) where applicable	NA
**Therapeutic Intervention**	9a	Types of therapeutic intervention (such as pharmacologic, surgical, preventive, self-care)	127
	9b	Administration of therapeutic intervention (such as dosage, strength, duration)	NA
	9c	Changes in therapeutic intervention (with rationale)	NA
**Follow-up and Outcomes**	10a	Clinician and patient-assessed outcomes (if available)	NA
	10b	Important follow-up diagnostic and other test results	92-97+128-129.
	10c	Intervention adherence and tolerability (How was this assessed?)	NA
	10d	Adverse and unanticipated events	NA
**Discussion**	11a	A scientific discussion of the strengths AND limitations associated with this case report	132-140
	11b	Discussion of the relevant medical literature **with references**.	141-145
	11c	The scientific rationale for any conclusions (including assessment of possible causes)	159-160
	11d	The primary "take-away" lessons of this case report (without references) in a one paragraph conclusion	165-169
**Patient Perspective**	12	The patient should share their perspective in one to two paragraphs on the treatment(s) they received	152-158
**Informed Consent**	13	Did the patient give informed consent? Please provide if requested	Yes ☑ No ☐
