# Supplementary figures and images for: Case report: A novel variant in SLC25A46 causing sensorimotor polyneuropathy and optic atrophy
Source: Front Neurol. 2022 Dec 12;13:1066040. doi: 10.3389/fneur.2022.1066040 (PMC9790965; doi:10.3389/fneur.2022.1066040)

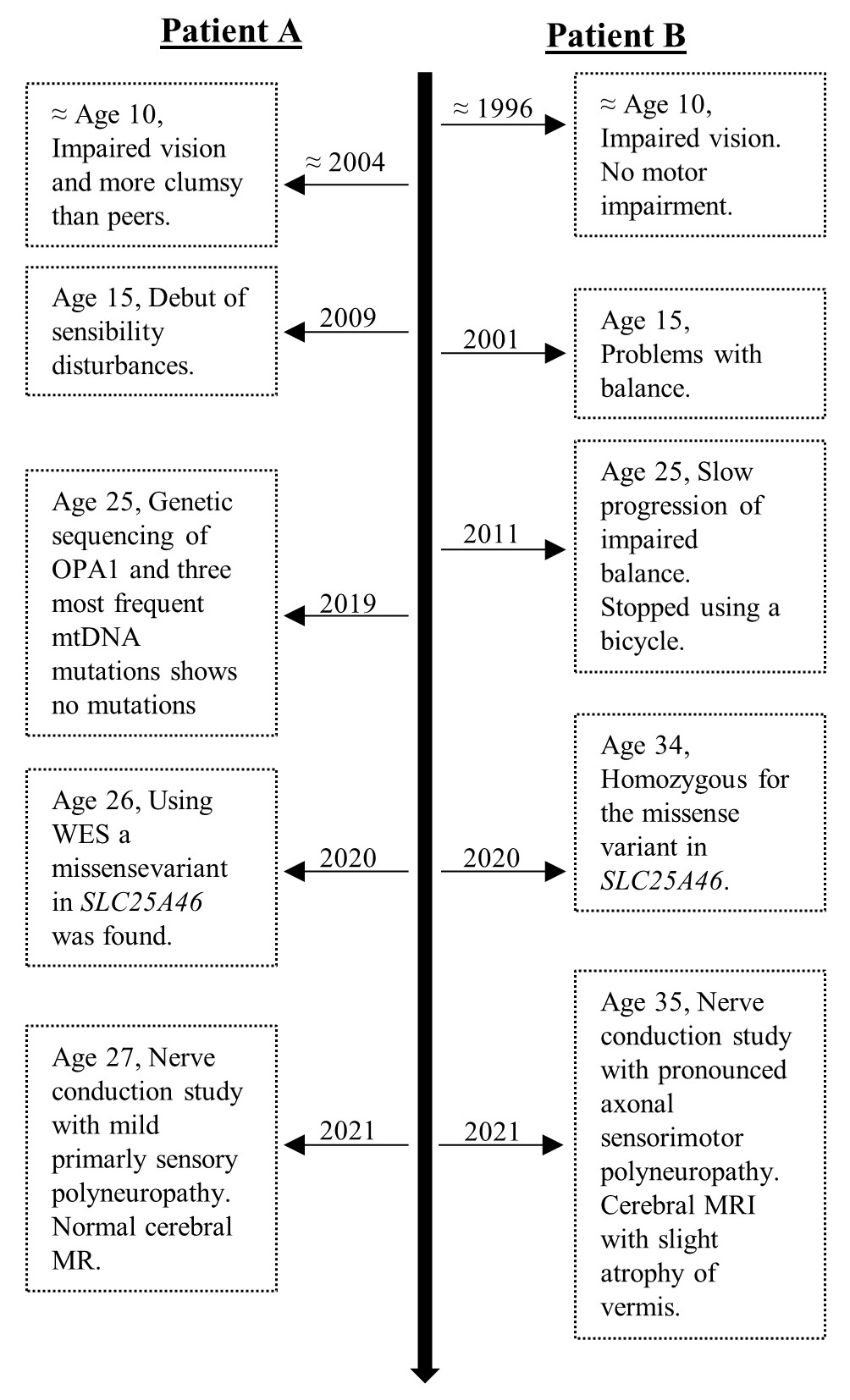

Supplement: Supplementary material 2 — Timeline of historical and current information for Patient A and Patient B. [file Image_1.JPEG]
